# Efficient analysis of time-to-event endpoints when the event involves a continuous variable crossing a threshold

**DOI:** 10.1016/j.jspi.2020.02.003

**Published:** 2020-09

**Authors:** Chien-Ju Lin, James M.S. Wason

**Affiliations:** a Medical Research Council Biostatistics Unit, University of Cambridge, UK; b Population Health Sciences Institute, Newcastle University, UK

**Keywords:** Phase II cancer trial, Progression-free survival, Longitudinal model

## Abstract

In many trials, the duration between patient enrolment and an event occurring is used as the efficacy endpoint. Common endpoints of this type include the time until relapse, progression to the next stage of a disease, or time until remission. The criteria of an event may be defined by multiple components, one or more of which may be a continuous measurement being above or below a threshold. Typical analyses consider all components as binary variables and record the first time at which the patient has an event. This is analysed through constructing and testing survival functions using Kaplan–Meier, parametric models or Cox models. This approach ignores information contained in the continuous components. We propose a method that makes use of this information to improve the precision of analyses using these types of endpoints. We use joint modelling of the continuous and binary components to construct survival curves. We show how to compute confidence intervals for quantities of interest, such as the median or mean event time. We assess the properties of the proposed method using simulations and data from a phase II cancer trial and an observational study in renal disease.

## Highlights

• Time-to-event outcomes sometimes involve continuous components crossing a threshold.
• Analyses typically consider the event as binary, discarding additional information.
• We propose an analysis method that uses the additional information.
• In some cases this dramatically improves the power and precision of the analysis.
• We show the utility of this on a cancer trial and a renal disease dataset.

## 1 Introduction

In clinical trials, it is common for the time until a patient relapses or progresses to the next stage of disease to be used as the endpoint assessing the efficacy of a new treatment. In many cases the event defining a progression or relapse (henceforth referred to as progression for simplicity) is defined by multiple criteria that must be met, one or more of which are continuous variables being above a threshold.

In this paper we consider two examples of such endpoints, although there are undoubtably many more (including HIV progression being defined by CD4 count or time until glycemic control in diabetes). The first example we consider is progression-free-survival (PFS), which is commonly used in solid tumour clinical trials. In PFS, the progression event is based on the Response Evaluation Criteria in Solid Tumours (RECIST) (Eisenhauer et al., 2009). A tumour progression for an individual patient is defined as either a 20% increase in the tumour size measurement from the minimum measurement observed or a new tumour lesion being observed.

The second example considered is an endpoint used in renal disease. In this case a progression is defined based on a decline in the estimated glomerular filtration rate (eGFR) beyond a certain threshold: unlike in oncology, the exact threshold varies between different studies.

Typical analyses of these endpoints involve fitting a model to the event or censoring times of all patients in the study. These event times are summarised by a survival function which contains information on the probability a patient survives until time t. There are three commonly used approaches to estimating the survival function. Firstly, there is the Kaplan–Meier (KM) survival function which is a non-parametric approach. The KM method treats time as discrete and hence results in a step function. It is based on the number of events and number of patients at risk at fixed points in time. The area under the survival curve is the mean survival time. Secondly, there are parametric survival models, which treat survival time as continuous and following a certain distribution such as exponential or Weibull. Finally, the Cox model is a semi-parametric approach, which uses explanatory variables to predict the hazard (i.e. instantaneous chance of an event). The Cox model can accommodate both discrete and continuous time.

The aforementioned methods construct survival functions based on events and non-events rather than considering the rich data collected on continuous measurements. Failure to use all information risks the loss of substantial efficiency (Dhani et al., 2009). In the context of analysing composite responder-based endpoints, researchers have proposed methods for utilising the continuous measurements to improve inference on the dichotomised binary outcome (Jaki et al., 2013, Karrison et al., 2007, Wason and Seaman, 2013, Lin and Wason, 2017). In particular, the augmented binary method, first proposed by Wason and Seaman (2013), estimates the proportion of patients who are responders, whilst using the continuous information to increase efficiency. The augmented binary method initially focused on estimating a probability of response at the end of two follow-up times. Lin and Wason (2017) extended the method to an arbitrary number of follow-up times as well as using the best observed response as an endpoint. The method has consistently been shown to provide extra precision on the estimated proportion of patients who are responders. It similarly can be used to increase the power to test for differences between arms in a randomised trial. However, the method has only focused on analysing a binary response outcome rather than the time until such an event occurs.

In this paper, we propose an augmented approach for improving inference on the time until a binary event, defined by underlying continuous measurements, occurs. We illustrate the methodology using simulations and real data from a real phase II cancer trial and a renal disease dataset.

## 2 Methods

In a study designed to assess time to event, patients are followed up until either an event occurs at a time G or a pre-planned time T. The maximum observed time is min(G,T). We assume that a progression event is defined by a composite outcome, comprising of both a continuous variable Y and a binary component D. No event occurring at time t can be written as $Y_{t}<c$ and $D_{t}=0$ where c is a threshold of response criteria and $Y_{t}$ and $D_{t}$ are observed values of Y and D at time t.

We first consider the basic framework that is used to model these data. The survival function, denoted by S(t), is the probability that a patient will have an event exceeding time t:S(t)=P(T>t)=1−P(T≤t). The hazard function, denoted by h(t), is, informally, the chance that the patient will experience an event in an instant of time t. The probability function, denoted by $P\left(Y_{t}<c\text{ and }D_{t}=0\right)$, is the unconditional probability that no events will occur at time t.

### 2.1 Kaplan–Meier estimator

The standard method of estimating the survival function was proposed by Kaplan and Meier (1958). The concept is to estimate the probabilities $P\left(Y_{t}<c\text{ and }D_{t}=0\right)$ for all the observed event times using the discrete event indicator. The survival function is then a product of the estimators of the probability function. We assume the data consist of n patients, with discrete event time t taking values in $\left\{t_{1},\ldots ,t_{T}\right\}$. Let $d_{m}$ be the number of events and $r_{m}$ be the number of patients at risk at time $t_{m}$. The Kaplan–Meier estimator of time t is defined as

$${\overset{ˆ}{S}}_{KM}\left(t\right)=\underset{t_{m}\leq t}{\prod }\left(1-\frac{\text{number of progression at }t_{m}}{\text{number of patients at risk at }t_{m}}\right)=\underset{t_{m}\leq t}{\prod }\left(1-\frac{d_{m}}{r_{m}}\right).$$

The variance of ${\overset{ˆ}{S}}_{KM}\left(t\right)$ is (Klein and Moeschberger, 2003 p. 105)

$$V\left({\overset{ˆ}{S}}_{KM}\left(t\right)\right)={\overset{ˆ}{S}}_{KM}{\left(t\right)}^{2}\sigma {\left(t\right)}^{2},\;\sigma {\left(t\right)}^{2}=\underset{t_{m}\leq t}{\prod }\left\{\frac{d_{m}}{r_{m}\left(r_{m}-d_{m}\right)}\right\}$$

The 95% confidence interval for the survival function for a single fixed time t is

$${\overset{ˆ}{S}}_{KM}\left(t\right)-Z_{1-\alpha ∕2}\sigma \left(t\right){\overset{ˆ}{S}}_{KM}\left(t\right),{\overset{ˆ}{S}}_{KM}\left(t\right)+Z_{1-\alpha ∕2}\sigma \left(t\right){\overset{ˆ}{S}}_{KM}\left(t\right),$$

where $Z_{1-\alpha ∕2}$ is 1−α∕2 percentile of a standard normal distribution.

The null hypothesis $H_{0}:S_{1}\left(t\right)=S_{2}\left(t\right)$ can be tested using a Chi-square test (Klein et al., 2007)

$$\chi_{1}^{2}=\frac{{\overset{ˆ}{S}}_{1}\left(t\right)-{\overset{ˆ}{S}}_{2}\left(t\right)}{{\overset{ˆ}{S}}_{1}{\left(t\right)}^{2}{\overset{ˆ}{\sigma }}_{1}{\left(t\right)}^{2}-{\overset{ˆ}{S}}_{2}{\left(t\right)}^{2}{\overset{ˆ}{\sigma }}_{2}{\left(t\right)}^{2}}.$$

A test for any difference between survival curves can be done by using a log-rank test. Rather than a direct comparison of those two rates, the log-rank test examines differences between observed and expected number of events at all observed times. Let $t_{1}<t_{2}<\cdots <t_{T}$ be the distinct event times in the pooled sample. Let $d_{.m}=d_{1m}+d_{2m}$ and $r_{.m}=r_{1m}+r_{2m}$ be the sum of the number of events and the number of patients at risk within each group at time $t_{m}$ of the two groups. The test statistic is

$$\chi_{1}^{2}=\frac{{\left(O_{1}-E_{1}\right)}^{2}}{E_{1}}+\frac{{\left(O_{2}-E_{2}\right)}^{2}}{E_{2}},$$

where $O_{1}$ and $O_{2}$ are the observed numbers of events at two groups, and $E_{1}$ and $E_{2}$ are the expected numbers of events, $E_{k}=\sum_{m=1}^{T}\frac{d_{.m}r_{km}}{r_{.m}}$.

### 2.2 Augmented binary method for time until a threshold event

#### 2.2.1 Single-arm

The continuous variable Y and binary component D are the two main components that define progression. If there are additional (continuous or non-continuous) components, one continuous component can be selected as Y and the remaining components combined into D.

A threshold c determines failure of the continuous variable Y. Without loss of generality we assume that Y>c means progression. We assume that D=1 means failure. The augmented binary method for solid tumour oncology (Lin and Wason, 2017) assumes (1) Y, the log tumour size ratio, follows a multivariate normal distribution and (2) D, the new-lesion progression indicator depends only on the observed tumour size at the previous visit. We consider how these assumptions can be relaxed and how sensitive results are to deviations when extending the method to time until progression.

We define $Y_{1},\ldots ,Y_{T}$ as the continuous component observations at each time and $D_{1},\ldots ,D_{T}$ as the binary component indicator at each time. It is assumed that patients are only followed until the event occurs and so values of $Y_{i}$ and $D_{i}$ after are not observed. $T_{Y}$ and $T_{D}$ are the time until $Y_{i}>c$ and $D_{j}=1$ respectively. The probability of no progression because of Y before time t, labelled $T_{Y}>t$ is:

(1) $$\begin{array}{cc} \text{Pr}\left(T_{Y}>t\right) & =\text{Pr}\left(Y_{1},\ldots ,Y_{t}\in \left(-\infty ,c\right),Y_{\left(t+1\right)},\ldots ,Y_{T}\in \left(-\infty ,\infty \right)\right)\\ & =\int_{-\infty }^{c}\ldots \int_{-\infty }^{c}f_{Y_{1},\ldots ,Y_{t}}\left(y_{1},\ldots ,y_{t};\theta \right)dy_{1}...dy_{t}. \end{array}$$

The value after time t is irrelevant to the probability in (1), represented by the integral limits being (−∞,∞).

The probability of no progression because of D before time t is

(2) $$\text{Pr}\left(T_{D}>t\right)=\text{Pr}\left(D_{1}=\cdots =D_{t}=0\right)$$

In Lin and Wason (2017), a logistic model is used to model the failure rate at the first time point and conditional logistic models for follow-up times, that is,

$$\text{Pr}\left(T_{D}>t\right)=\text{Pr}\left(D_{1}=0\right)\text{Pr}\left(D_{2}=0|D_{1}=0\right)\ldots \text{Pr}\left(D_{t}=0|D_{1}=\cdots =D_{t-1}=0\right)$$

However, there may be time points without a sufficient number of events for getting reliable estimators. In this situation, we use Cox regression to model the hazard function and transform it into the survival function. We do this as described below.

The hazard can be modelled by

$$h_{i}\left(t\right)=\lambda \left(t\right)\exp\left(\beta \prime x\right),$$

where i is the index of participant, λ(t) is the baseline hazard, x is a vector of explanatory variables associated with failure time, and β is a vector of regression coefficients. In the lesion progression model, the explanatory variables are the baseline tumour size and treatment indicator. $\overset{ˆ}{\lambda }\left(t\right)$ is estimated by Breslow’s estimator (Klein and Moeschberger, 2003 p. 283). Next, we use the relationship between S(t) and cumulative hazard H(t), S(t)=exp(−H(t)), to construct a survival function of new-lesion progression.

The final step is to aggregate Eqs. (1), (2). The event time of the patient i equalling or exceeding time t means that neither tumour progression nor lesion progression occurs before time t. The probability of no progression before time t can be written as

(3) $$S_{i}\left(t|\theta \right)=\text{Pr}\left(T_{i}=\min\left(T_{i,D},T_{i,Y}\right)\geq t\right)=\int_{\Omega^{t+k}}\text{Pr}\left(D_{1}=\cdots =D_{t}=0\right)f\left(y;\theta \right)dy,$$

where $\Omega =\left(-\infty ,c\right),k=\sum_{j=1}^{t}\left(j-1\right),y=\left(y_{1},\ldots ,y_{t}\right)$ and θ is the vector of parameters of above models. The average survival rate is $\overset{\sim }{S}\left(t|\theta \right)=\sum_{i=1}^{n}\frac{S_{i}\left(t|\theta \right)}{n}$ and is estimated by $\overset{\sim }{S}\left(t|\overset{ˆ}{\theta }\right)$. We use the delta method to construct a pointwise confidence interval for the survival function. Let $l\left(\theta \right)=\overset{\sim }{S}\left(t|\theta \right)$,

$$\text{Var}\left(l\left(\overset{ˆ}{\theta }\right)\right)\approx ▽{\left(l\left(\overset{ˆ}{\theta }\right)\right)}^{T}\text{Var}\left(\overset{ˆ}{\theta }\right)▽\left(l\left(\overset{ˆ}{\theta }\right)\right),$$

where θ is the vector of parameters. The delta method approximates the variance by a first-order Taylor expansion. Although this results in a straightforward estimator of the variance, we found there is an issue of estimators when near the boundary (i.e. when the survival function is near 1 or 0 — see Appendix A of the Supplementary Materials). To overcome this issue, we use the bootstrap (Davison and Hinkley, 1997) to estimate the variance near the boundary. A total of k bootstrap samples are generated from sampling the data with replacement to construct the distribution of $\overset{\sim }{S}\left(t|\overset{ˆ}{\theta }\right)$ at a fixed time t. From the bootstrap distribution, $\overset{\sim }{S}\left(t|{\overset{ˆ}{\theta }}_{1}\right),\ldots ,\overset{\sim }{S}\left(t|{\overset{ˆ}{\theta }}_{k}\right)$, we obtain the percentile, denoted by $\left({\overset{\sim }{S}}_{\alpha ∕2}^{*}\left(t|\overset{ˆ}{\theta }\right),{\overset{\sim }{S}}_{1-\alpha ∕2}^{*}\left(t|\overset{ˆ}{\theta }\right)\right)$, as the (1−α)% confidence interval. The drawback of the bootstrap confidence interval is its lengthy computational time.

The median survival time is defined as $M_{1i}=\inf\left(t:S_{i}\left(t\right)\leq 0.5\right)$. In some cases it may not be possible to estimate the median because there are not a sufficient number of events observed in the study. In that case it might make sense to estimate the mean survival time instead. The mean is estimated by the area under the $S_{i}\left(t\right)$ curve, which can be written as

$$M_{2i}=\overset{T_{i}}{\underset{t=1}{\sum }}tS_{i}\left(t|\theta \right).$$

#### 2.2.2 Comparative trials

We now extend the method to the problem of comparing the survival distribution of two treatments in a randomised trial. Both Wason and Seaman (2013) and Lin and Wason (2017) have addressed how to test for response rate at a fixed time. They add an arm indicator to models and apply the Wald test to test differences between mean response rates. We apply a similar strategy to test for S(t) of two arms at a fixed time. As for detecting differences between survival curves of two or more arms, we compare hazard rates. Let $R_{i}$ be the arm indicator for patient i, $R_{i}=0$ for control and 1 for experimental arms respectively. The continuous measurements are modelled by:

$${\left({\text{Y}}_{i1},{\text{Y}}_{i2},\ldots ,{\text{Y}}_{iT}\right)}^{\prime }|\text{R},z_{0}∼\text{N}\left({\left(\mu_{i1},\mu_{i2},\ldots ,\mu_{iT}\right)}^{\prime },\Sigma \right).$$

If logistic regression models are used for modelling the probability of D taking value 1 (e.g. death/lesion progression), the models could be written as:

(4) $$\text{logit}\left(p_{Di}\right)=\text{Pr}\left(D_{it}=1|D_{iu}=0\;\forall u\in \left\{1,\ldots ,\left(t-1\right)\right\};z_{i0},\ldots ,z_{iu}\right)=\alpha_{t}+\beta_{t}\text{R}+\gamma_{t}z_{i\left(t-1\right)}.$$

where $p_{Di}$ is the probability of patient i being a case of D failure at time t given that there is no D failure before time t. The parameter $\alpha_{t}$ represents the baseline log-odds of progression at time t. The parameter $\beta_{t}$ represents the log odds ratio of the experimental treatment.

If a Cox model is used for D, the hazard function can be written as

$$h_{i}\left(t|R\right)=\lambda \left(t\right)\exp\left(\beta_{1}z_{0i}+\beta_{2}R\right).$$

Based on the association between hazard function and survival function, we obtain

$$h_{i}\left(t|R\right)=H_{i}\left(t+1|R\right)-H_{i}\left(t|R\right),\;H_{i}\left(t|R\right)=-\log S_{i}\left(t|R\right),\text{ and }\text{Pr}\left(T_{D}>t\right)=\exp\left(-H_{i}\left(t|R\right)\right)$$

For a comparative trial, we test $H_{0}:h\left(t|R=0\right)=h\left(t|R=1\right)$ for all t≤τ, where τ is the largest time at which both groups have at least one patient at risk. An estimator of the expected hazard rate in the treatment arm under $H_{0}$ is the pooled estimator of the hazard rate, that is,

$$Z\left(\tau \right)=\overset{\tau }{\underset{t=1}{\sum }}\left(h\left(t|R=1\right)-\frac{h\left(t|R=0\right)+h\left(t|R=1\right)}{2}\right).$$

This will be close to zero if $H_{0}$ is true. Again, we calculate the variance by using either the delta or the bootstrap method. Let l(θ)=Z(τ), the variance using the delta method can be approximated by

$$\text{Var}\left(l\left(\overset{ˆ}{\theta }\right)\right)\approx ▽{\left(l\left(\overset{ˆ}{\theta }\right)\right)}^{T}\text{Var}\left(\overset{ˆ}{\theta }\right)▽\left(l\left(\overset{ˆ}{\theta }\right)\right),$$

where θ is the vector of parameters from the above models. The variance using the bootstrap method works as follows. A total of n bootstrap samples are generated from data with replacement. The value of Z of each sample is calculated and used to construct the bootstrap distribution of Z from which we obtain the variance. We will use Aug-delta to refer to the augmented binary method using the delta-method to estimate the variance and Aug-boot to refer to the method using the bootstrap variance. When the null hypothesis is true, $\frac{Z^{2}}{\text{Var}\left(Z\right)}$ has a chi-squared distribution with one degree of freedom. Given a significance level of α, $H_{0}$ is rejected when Z is larger than the upper α-level critical value of the $\chi_{1}^{2}$ distribution.

#### 2.2.3 Models for solid tumour oncology trials

As an illustration of the above framework, we show how it applies to progression-free survival in oncology.

RECIST (Eisenhauer et al., 2009) is used as a standard measurement of tumour response. Once lesions are identified, RECIST uses their longest diameter to define them being either “measurable” or “unmeasurable” lesions. Target lesions are selected from measurable lesions at baseline. The baseline and follow-up sum of the longest diameter of the target tumour lesions will then be used to assess tumour response. Henceforth we use the term “tumour size” to refer to the sum of the longest diameter of target tumour lesions. the term “progression” includes (1) increase in tumour size by more than 20% from a minimum (a tumour-growth progression) or (2) new lesions appearing (a new-lesion progression).

Let $z_{t}$ be the tumour size at time t, where t=0 refers to the baseline time. Let D be the new-lesion progression indicator where $D_{t}=0$ refers to no new-lesion progression between time (t−1) and t,(t=1,…,min(G,T)). The model assumptions are in line with Section 2.2.1. Considering all possibilities at which the minimum might occur at any of the T follow-up times, the log tumour size ratio for patient i is

$$\left(\log\frac{z_{i1}}{z_{i0}},\log\frac{z_{i2}}{\min\left(z_{i0},z_{i1}\right)},\ldots ,\log\frac{z_{iT}}{\min\left(z_{i0},\ldots ,z_{iT-1}\right)}\right).$$

For convenience, we eliminate the subscript i. The set of log tumour size ratios between all observed times can be written as $\left(Y_{10},Y_{20},\ldots ,Y_{T0},Y_{21},Y_{31},Y_{32},\ldots ,Y_{T\left(T-1\right)}\right)$, where $Y_{ab}$ is the ratio of log tumour size at time a and time b,

$$Y_{ab}=\log\frac{z_{a}}{z_{b}}=\log z_{a}-\log z_{b}+\log z_{0}-\log z_{0}=\log\frac{z_{a}}{z_{0}}-\log\frac{z_{b}}{z_{0}}.$$

Thus, $Y_{ab}$ is equivalent to $Y_{a0}-Y_{b0}$. Assuming log tumour size ratio from baseline follow a multivariate normal distribution, ${\left(Y_{10},Y_{20},\ldots ,Y_{T0},Y_{21},Y_{31},Y_{32},\ldots ,Y_{T\left(T-1\right)}\right)}^{\prime }$ can be written and modelled by

$${\left(Y_{10},Y_{20},\ldots ,Y_{T0},Y_{20}-Y_{10},Y_{30}-Y_{10},Y_{30}-Y_{20},\ldots ,Y_{T0}Y_{\left(T-1\right)0}\right)}^{\prime }∼N\left(A\mu^{T},A\Sigma A^{T}\right),$$

where

$$A=\left[\begin{array}{cccccccc} 1 & 0 & 0 & 0 & \ldots & 0 & 0 & 0\\ & & & \ddots & & & & \\ 0 & 0 & 0 & 0 & \ldots & 0 & 0 & 1\\ -1 & 1 & 0 & 0 & \ldots & 0 & 0 & 0\\ & & & \ddots & & & & \\ -1 & 0 & 0 & 0 & \ldots & 0 & 0 & 1\\ 0 & -1 & 1 & 0 & \ldots & 0 & 0 & 0\\ & & & \ddots & & & & \\ 0 & 0 & 0 & 0 & \ldots & 0 & -1 & 1 \end{array}\right].$$

The probability of no tumour-growth progression before time t is

$$\text{Pr}\left(T_{tumour}>t\right)=\int_{-\infty }^{\log\left(1.2\right)}\ldots \int_{-\infty }^{\log\left(1.2\right)}f_{Y_{10},\ldots ,Y_{t\left(t-1\right)}}\left(y_{10},\ldots ,y_{t\left(t-1\right)};\theta \right)dy_{10}...dy_{t\left(t-1\right)}.$$

The probability of no-lesion progression before time t is

$$\text{Pr}\left(T_{D}>t\right)=\text{Pr}\left(D_{1}=0\right)\text{Pr}\left(D_{2}=0|D_{1}=0\right)\ldots \text{Pr}\left(D_{t}=0|D_{1}=\cdots =D_{t-1}=0\right)$$

Again, the survival function can be obtained using Eq. (3).

## 3 Simulation study

### 3.1 Simulation strategy

In this section, we use simulated data to examine the performance of the proposed method. Simulation scenarios are designated for single-arm and comparative trials. Full details are given in Sections 3.1.1, 3.1.2. For the single arm case, we used “coverage” and “average width of the confidence intervals (CIs)” as measures of performance. Coverage is defined as the proportion of the 95% CIs that include the true survival rate. If two methods have similar coverage, the method with smaller average CI width is preferable due to increased precision. For comparative trials, we investigate the type I error rate, and power for detecting a difference between arms.

#### 3.1.1 Single-arm

We consider an endpoint based on tumour progression and simulate trials of 150 patients. The baseline tumour size of patients are generated from a U(0,1) distribution. Their log tumour size ratios (Y) are generated from a multivariate normal distribution with mean μ and covariance matrix Σ given below. The number of follow-up times is set to five for scenario 1 and seven for scenario 2. We define an event as tumour progression, and time to progression as the first time when a follow-up tumour size is greater than the minimum of all the previous records by more than 20%. For calculating the coverage, the actual survival rates in each scenario are estimated by using Kaplan–Meier estimators with $10^{5}$ participants.

• Scenario 1, five follow-up times, Y∼MVN(μ,Σ) where

$$\mu =\left(\begin{array}{c} 0\\ 0.036\\ 0.072\\ 0.108\\ 0.144 \end{array}\right)\;\;,\text{and }\Sigma =\left(\begin{array}{ccccc} 0.25 & 0.25 & 0.25 & 0.25 & 0.25\\ 0.25 & 0.45 & 0.45 & 0.45 & 0.45\\ 0.25 & 0.45 & 0.50 & 0.50 & 0.50\\ 0.25 & 0.45 & 0.50 & 0.75 & 0.75\\ 0.25 & 0.45 & 0.50 & 0.75 & 1 \end{array}\right).$$

• Scenario 2, seven follow-up times, we set $\mu ={\left(-0.22,-0.29,-0.36,-0.36,-0.43,-0.51,-0.36\right)}^{T}$ and $\sigma_{ii}=0.1,\sigma_{ij}=0.15,i\neq j;i,j=1,\ldots ,7$.

#### 3.1.2 Comparative trials

**Fig. 1:**
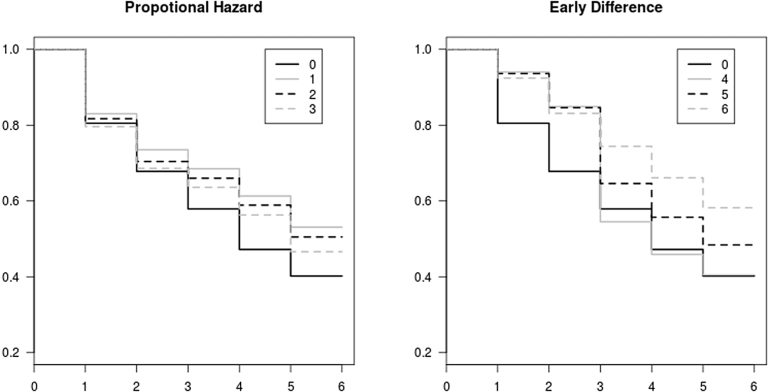
Survival functions of control and 6 treatment arms. The black solid line indicates control arm. The combinations of solid/dash and black/grey line are designed for scenarios 1 to 6. Results are based on the KM estimator with 1000 patients being randomly allocated to each arm. The parameters in each scenario are listed in Table 3.

Following the previous section, we consider both tumour progression and new-lesion progression. We simulate comparative trials with 200 patients allocated to each arm at random. The number of follow-up times is set to five. The actual survival rates of control and treatment arm under six scenarios are estimated by using Kaplan–Meier estimators with $10^{5}$ participants and are shown in Fig. 1. The solid black line indicates the control arm, labelled 0. The combinations of solid/dash and black/grey lines indicate survival functions of the treatment arm under different scenarios, labelled 1–6. The different scenarios represent potential possibilities of the difference between experimental and control arms’ survival functions, including proportional hazards (PH), an early difference, a late difference, and crossing of survival functions. The models from which log tumour size and new-lesion progression are generated are described in the following paragraphs.

*Tumour progression* We assume that patients are followed up for five time points. The mean log tumour size ratios of the control arm are generated from a multivariate normal with mean μ+0.1 and covariance matrix Σ, where

$$\mu =\left(\begin{array}{c} -0.2\\ -0.4\\ -0.56\\ -0.6\\ -0.65 \end{array}\right)\;\;,\text{and }\Sigma =\left(\begin{array}{ccccc} 0.05 & 0.05 & 0.05 & 0.05 & 0.05\\ 0.05 & 0.10 & 0.10 & 0.10 & 0.10\\ 0.05 & 0.10 & 0.14 & 0.14 & 0.14\\ 0.05 & 0.10 & 0.14 & 0.16 & 0.16\\ 0.05 & 0.10 & 0.14 & 0.16 & 0.18 \end{array}\right).$$

These numbers are based on data from HORIZON II (described further in Section 4.1). The values of μ in scenarios 1–6 are listed in the second column of Table 3. We calculate the time to tumour progression based on the generated tumour size ratios.

*New-lesion progression* (Bender et al., 2005) derived a general formula to address the relationship between the hazard and the corresponding survival time which can be used to generate survival times to simulate Cox proportional hazards. The concept is that since the survival function S is decreasing and continuous, then the inverse function $S^{-1}\left(v\right),v\in \left[0,1\right]$ has a unique time t such that S(t)=v. The association between survival time and hazard, S(t)=exp(−H(t)), implies $T=S^{-1}\left(V\right)=H^{-1}\left(-\frac{\log\left(V\right)}{\lambda \exp\left(x^{\prime }\beta \right)}\right)$ where the random variable V follows a uniform distribution on (0,1).

We use this formula and simulate time to new-lesion progression from an exponential regression model. The hazard model can be written as

$$H\left(t,x\right)=\lambda \exp\left(x^{\prime }\beta \right),$$

where λ is the baseline hazard and x is the covariate. If the hazard is proportional, the hazard ratio for control and treatment is constant over time, and equal to $e^{\beta }$.

A choice of λ=0.1 is equivalent to a constant new-lesion failure rate of 10% at each time point. A negative value of β means that the treatment arm has a lower rate of new lesion progressions. The generated time to new-lesion progression is rounded up to the nearest time. This is to reflect that in reality progressions are interval censored. The parameters of (λ,β) of control arm is (0.1, 0), and (λ,β) of scenarios 1–6 are listed in Table 3.

### 3.2 Simulation results

Table 1 shows the results from 1000 replicates of a single-arm trial using the Aug-boot and the Kaplan–Meier method (KM). The true S(t) of the five time points are 0.644, 0.394, 0.276, 0.160, 0.096. The results show that the estimators of both methods are close to unbiased. The two methods have similar coverage (Aug-boot is around 96% and KM is 95%), but the average CI width of Aug-boot is narrower than that of KM. This suggests that the Aug-boot gives extra precision compared to KM. Similar results are found in scenario 2 with seven follow-up times (see Table 2).

Table 3 shows the results from comparative trial simulations using the log-rank test, Peto & Peto, and the Aug-delta method. The empirical Type I error of the three methods is 0.046, 0.044, and 0.056, respectively. The last three columns show the power to detect differences between control and experimental arms in scenarios 1–6. The scenarios represent: proportional hazards in new-lesion progression (scenarios 1,2,3); difference in tumour progression in early time but the reverse at late time (scenario 4); difference in early time (scenario 5); difference at all 5 follow-up time points (scenario 6). The power of all three methods increase as β (hazard ratio) increases. Aug-delta provides a big gain in power in some scenarios such as crossing survival functions and difference in early time. In other scenarios it provides a more moderate gain such as in scenarios 1 and 2. In a couple of scenarios it actually had a lower power, especially so for scenario 6.

**Table 1:** Estimated survival function and coverage of the augmented binary method with bootstrap (Aug-boot) in comparison with Kaplan–Meier estimator (KM) which dichotomises continuous variables. Results are based on 1000 iterations.

Time	True	S(t)		Estimated coverage		Average CI width	
		KM	Aug-boot	KM	Aug-boot	KM	Aug-boot
1	0.644	0.641	0.642	0.936	0.965	0.153	0.138
2	0.394	0.391	0.391	0.959	0.964	0.157	0.134
3	0.276	0.275	0.276	0.941	0.965	0.144	0.118
4	0.160	0.161	0.162	0.951	0.965	0.120	0.090
5	0.096	0.096	0.097	0.956	0.964	0.097	0.065

**Table 2:** Estimated survival function with up to seven follow-up times and coverage of the augmented binary method with bootstrap (Aug-boot) in comparison with Kaplan–Meier estimator (KM) which dichotomises continuous variables.

Time	True	S(t)		Estimated coverage		Average CI width	
		KM	Aug-boot	KM	Aug-boot	KM	Aug-boot
1	0.852	0.8644	0.8568	0.966	0.978	0.109	0.095
2	0.636	0.6377	0.6371	0.979	0.981	0.154	0.137
3	0.452	0.4182	0.4235	0.982	0.984	0.159	0.121
4	0.271	0.2547	0.2688	0.978	0.982	0.141	0.089
5	0.177	0.1732	0.1615	0.976	0.982	0.124	0.072
6	0.119	0.1111	0.1112	0.98	0.983	0.103	0.053
7	0.043	0.0453	0.0425	0.984	0.984	0.072	0.024

**Table 3:** Power of detecting differences from control arm using the log-rank test (KM estimator), Peto & Peto modification of the Gehan–Wilcoxon test, augmented binary method with delta method (Aug-delta). The first four columns show the parameters of models for control and scenarios 1 to 6. The scenarios 1 to 3 are designed with proportional hazards, and scenarios 4, 5, 6 are designed with differences between mean log tumour size ratio. Note that there is an early difference between the survival curves of scenario 4 and the control, but that they are slightly overlap at the last two time points (see Fig. 1).

Scenario	Note	μ	β	λ	Log-rank	Peto	Aug-delta
0	Control	−0.10 −0.30 −0.46 −0.50 −0.55	0	0.1	–	–	–
–	No diff	−0.10 −0.30 −0.46 −0.50 −0.55	0	0.1	0.046	0.044	0.056
1	PH	−0.10 −0.30 −0.76 −0.80 −0.85	−0.5	0.1	0.732	0.675	0.800
2	PH	−0.10 −0.30 −0.76 −0.80 −0.85	−0.3	0.1	0.491	0.426	0.520
3	PH	−0.10 −0.30 −0.76 −0.80 −0.85	−0.1	0.1	0.199	0.168	0.178
4	Crossing	−0.50 −0.70 −0.63 −0.67 −0.72	0	0.05	0.096	0.234	0.840
5	Early diff	−0.40 −0.60 −0.61 −0.65 −0.70	0	0.05	0.604	0.762	0.927
6	Diff over time	−0.30 −0.50 −0.66 −0.70 −0.75	0	0.05	0.980	0.990	0.942

## 4 Case study

### 4.1 Application to HORIZON II trial

HORIZON II (clinicaltrials.gov identifier: NCT00384176) is a three arm colon cancer trial sponsored by AstraZeneca. Patients recruited in the first part of the trial were randomly assigned 1:1:1 to placebo, cediranib 20 mg once daily, cediranib 30 mg once daily; after an interim analysis subsequent patients were randomly assigned 1:2 to placebo or cediranib 20 mg (Hoff et al., 2012). The number of patients with baseline and at least one follow-up record for the three arms are 331, 457, 192, respectively. Their tumour sizes were measured every six weeks up to 24 weeks and then every 12 weeks until progression.

The endpoint is progression free survival (PFS) after a year. PFS is defined as the time from the date of randomisation to the date of death from any cause or tumour/lesion progression. Figure A.1 in supplementary material shows the tumour size ratio of patients from baseline to different post-baseline timepoints. The colours from dark grey to light grey denote tumour size ratios from 0.01 to 1.199. Tumour progression, lesion progression or both are indicated by light blue, blue, and dark blue, respectively. In addition, the colours white and green indicate, respectively, no data is observed and complete response (100% of shrinkage). The normality assumption appears to hold reasonably well as shown in the supplementary material of Lin and Wason (2017).

No data being observed can be caused by either a missed clinic visit or the patient completely dropping out from the trial. We first consider missed clinic visits. There are cases where patients have a follow-up observation at time $t_{m}$ but no observations at time $t,t<t_{m}$. It is possible that if a progression occurred at time $t_{m}$ it may have actually occurred earlier, leading to interval censored data. Since the Aug-delta method estimates the probability of no progression by integrating functions of the constructed model over all possible tumour size ratio for missing clinic visits, it accounts for missed clinic visits in its inference. We next consider dropout, which results in right-censoring. As shown in Figure A.1, the average dropout rates in the placebo, cediranib 20 mg, cediranib 30 mg arms are 16.4%, 12.5% and 13.8% respectively. The Aug-delta uses the model to deal with censoring. It estimates the probabilities at each follow-up time which accounts for censored patients. This would be similar to making the assumption that censoring is non-informative, although has the advantage that the missing data in each component can be accounted for using a missing at random assumption (i.e. additional covariates could be included in the separate components of the model).

Table 4 shows the result of comparison between placebo, 20 mg group and 30 mg group using logrank test and restricted mean survival time (RMST) (Royston and Parmar, 2013). The efficiency of test using RMST has been discussed (Tian et al., 2017). The restricted mean is estimated as the area under the survival curve up to 5 follow-up times. As seen, the standard error of the difference in RMST estimated from Aug-delta is smaller than the difference in RMST estimated from non-parametric method. The log-rank test shows that the survival curves between placebo and cediranib 20 mg is different (p = 0.0002) and the survival curves between placebo and cediranib 30 mg is also different (p = 0.0095). The Aug-delta gives the same conclusion about cediranib 20 mg (p = 0.0125) and cediranib 30 mg (p = 0.0006).

**Table 4:** Logrank test, restricted mean survival time test between placebo, 20 mg group and 30 mg group using KM and Aug-delta.

	KM	Aug-delta
RMST in Placebo $\left({\overset{ˆ}{\mu }}_{1}\right)$	4.23	3.70
RMST in 20 mg $\left({\overset{ˆ}{\mu }}_{2}\right)$	4.45	3.90
RMST in 30 mg $\left({\overset{ˆ}{\mu }}_{3}\right)$	4.39	4.08
Diff. ${\overset{ˆ}{\Delta }}_{2}={\overset{ˆ}{\mu }}_{2}-{\overset{ˆ}{\mu }}_{1}$ (SE)	0.22 (0.086)	0.20 (0.014)
Diff. ${\overset{ˆ}{\Delta }}_{3}={\overset{ˆ}{\mu }}_{3}-{\overset{ˆ}{\mu }}_{1}$ (SE)	0.16 (0.106)	0.38 (0.027)
P-value for ${\overset{ˆ}{\Delta }}_{2}$ (RMST test)	0.0053	<0.0001
P-value for ${\overset{ˆ}{\Delta }}_{3}$ (RMST test)	0.1458	<0.0001
P-value for HR-Placebo and 20 mg (logrank test)	0.0002	0.0125
P-value for HR-Placebo and 30 mg (logrank test)	0.0095	0.0006

RMST: Restricted mean survival time.

### 4.2 Application to pre-End-Stage Renal Disease (pre-ESRD) study

A study of chronic kidney disease (CKD) patients receiving multidisciplinary care was conducted by the China Medical University in Taiwan between 2003 and 2015 (Tsai et al., 2018). They concluded that changes of the estimated glomerular filtration rate (eGFR) at the first year of pre-End-Stage Renal Disease (pre-ESRD) programme enrolment were factors associated with developing ESRD. Some research has been done on the progression of CKD. However, the progression of kidney function is vaguely defined. Tsai et al. (2017) defined the CKD progression as an annual eGFR decline rate over 3 ml/min/1.73 m^2^. Two of the recommended definitions of CKD progression by the Kidney Disease: Improving Global Outcomes CKD Work Group (KDIGO) (Kidney Disease: Improving Global Outcomes CKD Work Group, 2013) were (1) a decrease of 25% in eGFR from baseline and (2) rapid progression: a sustained decline in eGFR of > 5 ml/min/1.73 m^2^ /year from baseline.

In this application, we define progression as an annual eGFR decline rate (from baseline) over 25%. The baseline of eGFR is defined as the eGFR of the date when the patient entered pre-ESRD or a day before. If no observations are available for both days, the first eGFR record after pre-ESRD enrolment is used. The outcome is time to progression, defined as the time from the date of pre-ESRD enrolment to the date of progression. There are no cases of death during the first year in this dataset. Patients were followed up either every three months or every month depending on their CKD severity. On average, the interval was three months. Therefore, we defined M1, M2, M3, M4 as representing the intervals 1–3 months, 4–6 months, 7–9 months and 10–12 months. For multiple records within each time interval, the average eGFR is used. Patients without any eGFR records from M1 to M4 were excluded.

Figure A.2 in supplementary material demonstrates that the distribution of ratio of follow-up eGFR to baseline deviates from normality; we therefore applied the best fitting Box–Cox transformation (Box and Cox, 1964), which was $\frac{y^{-0.3}-1}{-0.3}$. For missed clinic visits, the Aug-delta method estimates probability of progression for missed visits prior to the last observation. We felt there is a need for further exploration of analysis of missing records. Therefore, we re-defined the length of interval. $\left(x_{1},\ldots ,x_{4}\right)$ as the ratio of eGFR at time $t_{1},\ldots ,t_{4}$. If $x_{i}:t_{m}\leq t_{i}<t_{s}$ is missing and $x_{s}<0.75$, we assume the progression should occur any time from $t_{m}$ to $t_{s}$. $x_{s}$ represents the record observed at the interval of $\left(t_{m},t_{s}\right)$. We denote the analysis using the KM method on the re-defined intervals by ${\text{KM}}^{+}$. Table 5 shows the estimated survival of 4534 patients using the KM, ${\text{KM}}^{+}$ and the Aug-delta and the reduction in 95% confidence interval (CI) from KM to Aug-delta, $\frac{\text{CI width of Aug-delta - CI width of KM}}{\text{CI width of KM}}$. Results show that the estimated PFS using ${\text{KM}}^{+}$ is smaller than the KM method but closer to Aug-Delta and the Aug-delta gives a considerably smaller 95% CI than the KM method.

**Table 5:** Survival function and 95% confidence interval of Pre-ESRD- CKD progression using KM method and Aug-delta. As there are cases that patients have records at time ${\text{M}}_{t}$ but missing records at ${\text{M}}_{t-1}$, we treat it as interval censored where interval is $\left({\text{M}}_{t-1},{\text{M}}_{t}\right)$. This means if a patient has a progression at M3 but missing record at M2. They would be treated as having progression in (M2, M3), instead of assuming no progression at M2.

Time	Mean of estimated survival			CI width		$\frac{\text{CI width of Aug-delta - CI width of KM}}{\text{CI width of KM}}$
	KM	${\text{KM}}^{+}$	Aug-Delta	KM	Aug-Delta	
M1	0.936	0.936	0.940	0.014	0.007	0.508
M2	0.866	0.853	0.859	0.020	0.013	0.356
M3	0.800	0.778	0.768	0.023	0.018	0.244
M4	0.736	0.708	0.664	0.026	0.021	0.171

${}^{+}$ change the interval censored width.

## 5 Discussion

In this paper we considered how one can efficiently analyse a time-to-event endpoint when the event is formed from a continuous measurement being above or below a threshold. We consider a method that allows the continuous information to be used in order to improve precision. This method is an extension of the method of Lin and Wason (2017) from a response-based endpoint to progression-free survival. We showed its potential through two real data applications: firstly in a solid tumour oncology trial, and secondly in an observational study of renal disease.

We have showed through simulation and real data that the proposed method can provide substantial extra precision in single-arm trials and more power for randomised two-arm trials. Unlike earlier work proposing a similar approach for analysing response outcomes (Wason and Seaman, 2013, Lin and Wason, 2017), we did not find that the proposed method was always more powerful. Instead, the power gain seems highly variable. In some scenarios the power gain was huge, such as when there were crossed survival functions or large differences in the hazard functions earlier on. In other cases the proposed method actually lost (generally a modest amount of) power compared to standard analysis approaches. This indicates the proposed method may be suitable as a secondary analysis rather than replacing standard time-to-event methods as a primary analysis.

There are some issues to be considered before using the augmented method. First of all, time to actual progression. In the renal disease study, patients were followed for 10 years during which they received medication when progression was “observed”. Once progression occurred, they would be followed up more frequently for a certain period. Time to the first progression or time from the first progression to the second progression is often the focus of the research. A missing record might result in biased estimation, that is, if a patient does not have a record at time $t_{m}$ but has a record at $t_{m+1}$, the KM method treats the patient as if there is no progression at $t_{m}$. In other words, the KM method imputes missing observations as no progression. This may result in the survival rate at $t_{m}$ being overestimated. In contrast, the augmented binary method uses predicted probability as if the patient was followed up at $t_{m}$ to deal with missing/censoring cases. This probability can be interpreted as a weighted probability of the occurrence of progressions. Dichotomised variables take a value of either 0 or 1, whereas the augmented binary method takes the weighted probability of patients with similar records having no progression. For a study where the time to actual progression is of interest, we would argue that the augmented binary method is estimating a more correct quantity than the KM method.

The second concern is the computational time. Multi-dimensional integration in the augmented binary method adds to the computational burden. The bootstrap method, Aug-boot, has advantages when survival probabilities are close to the boundary but requires substantial additional computation. For example, in the simulation study with five follow-up times and sample size 50, when we increased the number of bootstrap samples from 30 to 100, the computational time for one iteration increased from 20 min to an hour. Some of the increased computational burden may be addressed through utilising parallel computation. When applying the delta method, the numerical partial derivatives could be paralellised. If applying a bootstrap procedure then the bootstrap samples could be parallelised. Lastly, if conducting simulation studies to assess statistical properties, then replicates could be parallelised.

The third concern is the cost of investigating a more complex model. This model makes additional assumptions that the traditional methods do not. One important such assumption is normality of residuals in the model for the continuous variables. Previous work (Wason and Seaman, 2013, McMenamin et al., 2019) has demonstrated in similar situations that results can be sensitive to this assumption. It is therefore very important to investigate this assumption; if the normality assumption does not appear to hold then a transformation such as the family of Box and Cox may be sufficient to improve the properties. In the applications, the normality assumption was met for the log tumour-ratio in the HORIZON II study. In renal disease dataset, we defined progression using the relative difference. The multivariate normal model fitted the Box–Cox transformed ratio well. In some cases it may be difficult to find a suitable transformation, such as if the continuous variable is zero-inflated. Further work is motivated to allow for more general distributions for modelling the underlying continuous data. This would lead to more robust conclusions when the multivariate normal does not fit well to any transformation.

Lastly the proposed model allows for both interval- and right-censoring through the individual components of the joint model. We have explained how this is done within the case studies but further investigation of the effect of informative censoring would be of interest. We hypothesise that the proposed methods would not be any more sensitive to non-informative censoring than any other standard time-to-event analysis and may actually be less sensitive to informative censoring, but this requires some investigation. We note in some cases individual components of the event variable may be missing, and this is a situation that our approach is likely to deal with better.

We have applied the augmented binary method to oncology and kidney disease. However, the proposed method is not limited to these. The concept can be applied to any case where progression criteria involve multiple variables, at least one of which is continuous.

## CRediT authorship contribution statement

**Chien-Ju Lin:** Conceptualization, Methodology, Software, Formal analysis, Writing - original draft, Visualization. **James M.S. Wason:** Conceptualization, Methodology, Writing - review & editing, Supervision, Funding acquisition.

## Appendix A Supplementary data

MMC S1 Supplementary material (additional methods on finding the coverage probability, additional simulation results on the coverage, and visualisation of the HORIZON dataset).
