# Supplementary material for: Efficient analysis of time-to-event endpoints when the event involves a continuous variable crossing a threshold
Source: J Stat Plan Inference. 2020 Sep;208:119–29. doi: 10.1016/j.jspi.2020.02.003 (PMC7097971; doi:10.1016/j.jspi.2020.02.003)
Supplement: MMC S1 — Supplementary material (additional methods on finding the coverage probability, additional simulation results on the coverage, and visualisation of the HORIZON dataset). [file mmc1.pdf]

Efficient analysis of time-to-event endpoints when the event involves a continuous variable crossing a threshold : Supplementary Materials

CHIEN-JU LIN and JAMES WASON

A.1 Coverage probability of delta method

We consider one follow-up time and assume that Y follows a normal distribution with mean μ and variance σ^2. The cumulative function is written by

$$F(r) = \int_{-\infty}^{r} \frac{1}{\sqrt{2\pi}\sigma} e^{-\frac{1}{2\sigma^2}(y-\mu)^2} dy$$

(1) Theoretical derivative

$$\begin{aligned}
\frac{d}{d\mu}F(r) &= \frac{d}{d\mu}\int_{-\infty}^{r} \frac{1}{\sqrt{2\pi}\sigma} e^{-\frac{1}{2\sigma^2}(y-\mu)^2} dy = \int_{-\infty}^{r} \frac{1}{\sqrt{2\pi}\sigma} e^{-\frac{1}{2\sigma^2}(y-\mu)^2} \frac{d}{d\mu}\Bigg[-\frac{1}{2\sigma^2}(y-\mu)^2\Bigg] dy \\
&= \int_{-\infty}^{r} \frac{1}{\sqrt{2\pi}\sigma} e^{-\frac{1}{2\sigma^2}(y-\mu)^2} \frac{1}{\sigma^2}(y-\mu) dy \\
&= \int_{-\infty}^{z(r)} \frac{1}{\sqrt{2\pi}\sigma} e^{-z} dz \text{ , where } z = \left(\frac{y-\mu}{\sigma}\right)^2 \\
&= -\int_{z(r)}^{\infty} \frac{1}{\sqrt{2\pi}\sigma} e^{-z} dz \\
&= \frac{1}{\sqrt{2\pi}\sigma} e^{-z}|_{z(r)}^{\infty} \\
&= -\frac{1}{\sqrt{2\pi}\sigma} e^{-z(r)} = -f(r) \text{ , where f(.) is the pdf of y.}
\end{aligned}$$

(2) Numerical differentiation

$$F'(r,\mu) = \lim_{h=\rightarrow 0} \frac{F(r,\mu+h) - F(r,\mu)}{h}$$

Table A.1: coverage of using delta method with theoretical and numerical differentiation for various (μ, σ)

	$(\mu = 0, \sigma = 1)$		$(\mu = 0, \sigma = 0.5)$	
r	Numerical	Theoretical	Numerical	Theoretical
log(0.7)=-0.36	0.944	0.944	0.925	0.925
log(0.8)=-0.22	0.932	0.932	0.928	0.928
log(0.9)=-0.11	0.945	0.945	0.953	0.953
0	0.94	0.94	0.944	0.944
log(1.1)=0.10	0.949	0.949	0.94	0.94
log(1.2)=0.18	0.944	0.944	0.931	0.931
	$(\mu = -0.5, \sigma = 1)$		$(\mu = -0.5, \sigma = 0.5)$	
r	Numerical	Theoretical	Numerical	Theoretical
-0.36	0.964	0.964	0.954	0.954
-0.22	0.924	0.924	0.942	0.942
-0.11	0.945	0.945	0.926	0.926
0	0.928	0.928	0.914	0.914
0.10	0.932	0.932	0.9	0.9
0.18	0.92	0.92	0.903	0.903

$$h(A) \approx h(B) + \nabla h(B)^T (A - B)$$

$$h(A) \approx h(B) + \nabla h(B)^T (A - B) + \frac{(\nabla)^2}{2} h(B)^T (A - B)^2$$

$$\begin{aligned} var(h(A)) &\approx var(h(B) + \nabla h(B)^T (r - B) + \frac{(\nabla)^2}{2} h(B)^T (r - B)^2) \\ &\approx \nabla h(B)^T cov(A) \nabla h(B) + \frac{1}{4} (h'')^2 (B) var(A^2) + (h'')^2 var(A) B^2 \end{aligned}$$

We use delta method to obtain $var(F)$, that is,

$$var(F(\hat{\beta})) \approx \nabla F(\hat{\beta})^T var(\hat{\beta}) \nabla F(\hat{\beta})$$

Define coverage probability as the proportion of the 95 % confidence interval cover true F(r). Table A.1 shows the coverage probability for various (μ, σ, r). The Result is based on 1000 iterations and h is set to 0.0001. As seen, the result of using theoretical directive and numerical differentiation are the same. The coverage goes worse when predicted probability close to boundary

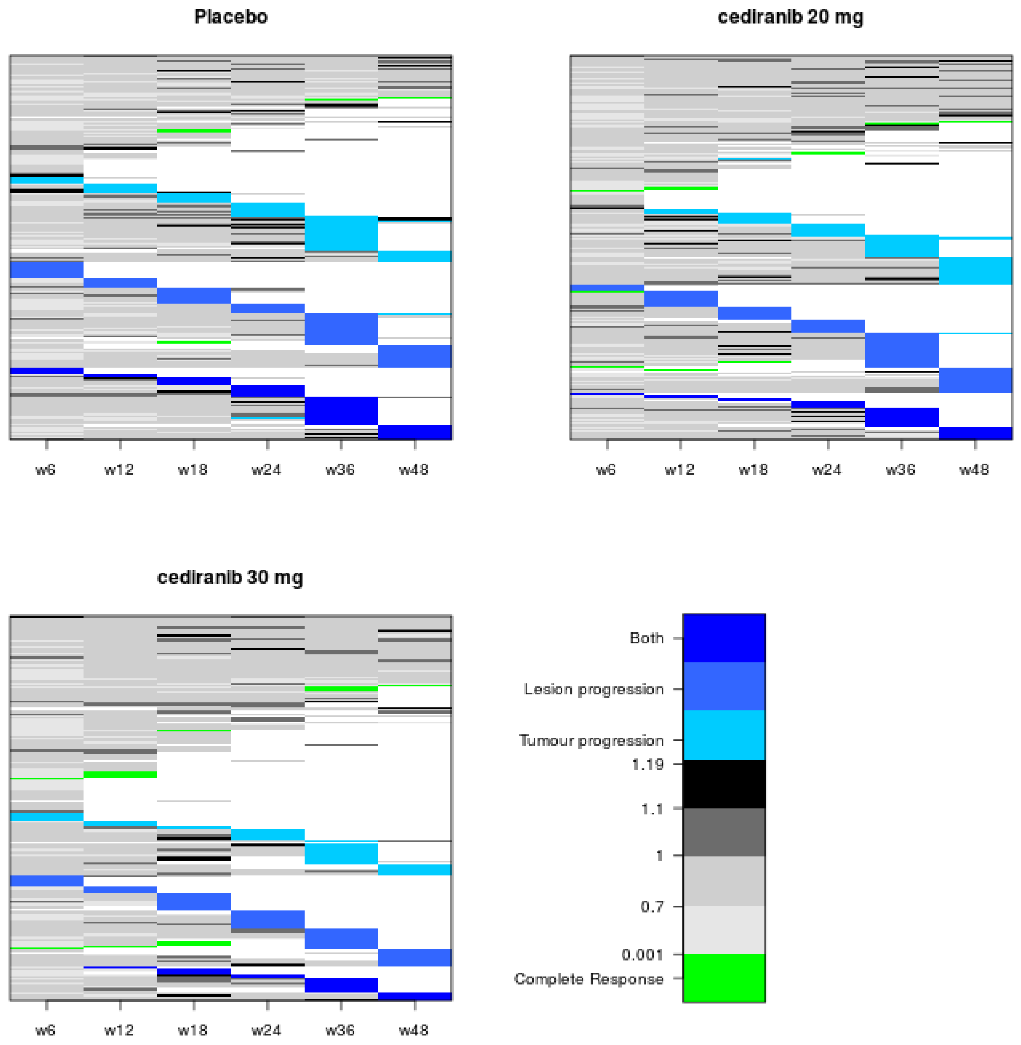

Figure A.1: Tumour size ratios from the cumulative minimum of patients and their progression status.

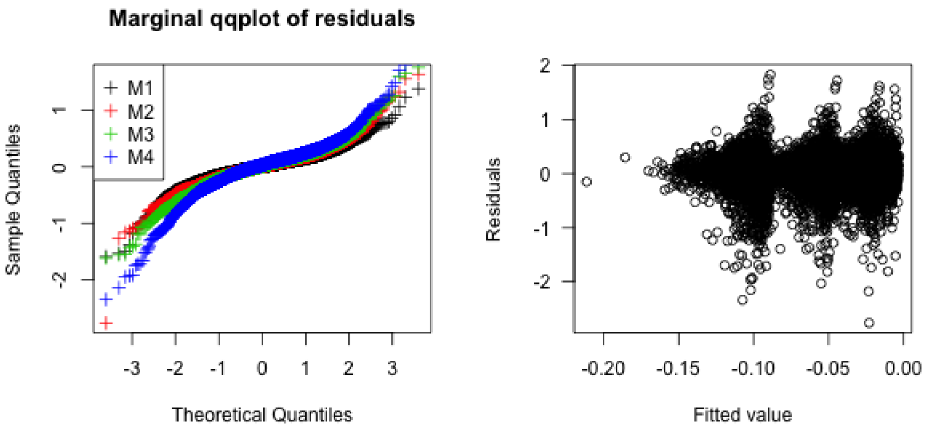

Figure A.2: QQ- and fitted values vs residual plots from renal dataset.
